# Visualizing Diverse RNA Functions in Living Cells With Spinach^TM^ Family of Fluorogenic Aptamers

**DOI:** 10.21769/BioProtoc.5504

**Published:** 2026-06-20

**Authors:** Ryan O’Hanlon, Karen Y. Wu

**Affiliations:** Lucerna, Inc, Brooklyn, NY, USA

**Keywords:** RNA imaging, Fluorescence microscopy, Fluorogenic RNA, Single-molecule resolution, Live-cell imaging, Transcription, RNA degradation, RNA aggregation

## Abstract

RNA is now recognized as a highly diverse and dynamic class of molecules whose localization, processing, and turnover are central to cell function and disease. Live-cell RNA imaging is therefore essential for linking RNA behavior to mechanism. Existing approaches include quenched hybridization probes that directly target endogenous transcripts but face delivery and sequestration issues, protein-recruitment tags such as MS2/PP7 that add large payloads and can perturb localization or decay, and CRISPR–dCas13 imaging that requires substantial protein cargo and careful control of background and off-target effects. Here, we present a protocol for live-cell RNA imaging using the Spinach^TM^ family of fluorogenic RNA aptamers. The method details the design and cloning of Spinach^TM^-tagged RNA constructs, selection and handling of cognate small-molecule fluorophores, expression in mammalian cell lines, dye loading, and image acquisition on standard fluorescence microscopes, followed by quantitative analysis of localization and dynamics. We include controls to verify aptamer expression and signal specificity, guidance for multiplexing with related variants (e.g., Broccoli, Corn, Squash, Beetroot), and troubleshooting for dye permeability and signal optimization. Application examples illustrate use in tracking cellular delivery of mRNA therapeutics, monitoring transcription and decay in response to perturbations, and the forming of toxic RNA aggregates. Compared with prior methods, Spinach^TM^ tags are compact, genetically encodable, and fluorogenic, providing high-contrast imaging in both the nucleus and cytoplasm with single-vector simplicity and multiplexing capability. The protocol standardizes key steps to improve robustness and reproducibility across cell types and laboratories.

Key features

• This protocol demonstrates the usage of the Spinach^TM^ technology in different RNA-focused applications.

• The protocol can be used to fluorescently image most RNA types in live cells.

• This protocol requires pre-existing experience in molecular cloning, cell culturing, and fluorescence microscopy.

• Requires at least seven days to complete from beginning to end.

## Graphical overview


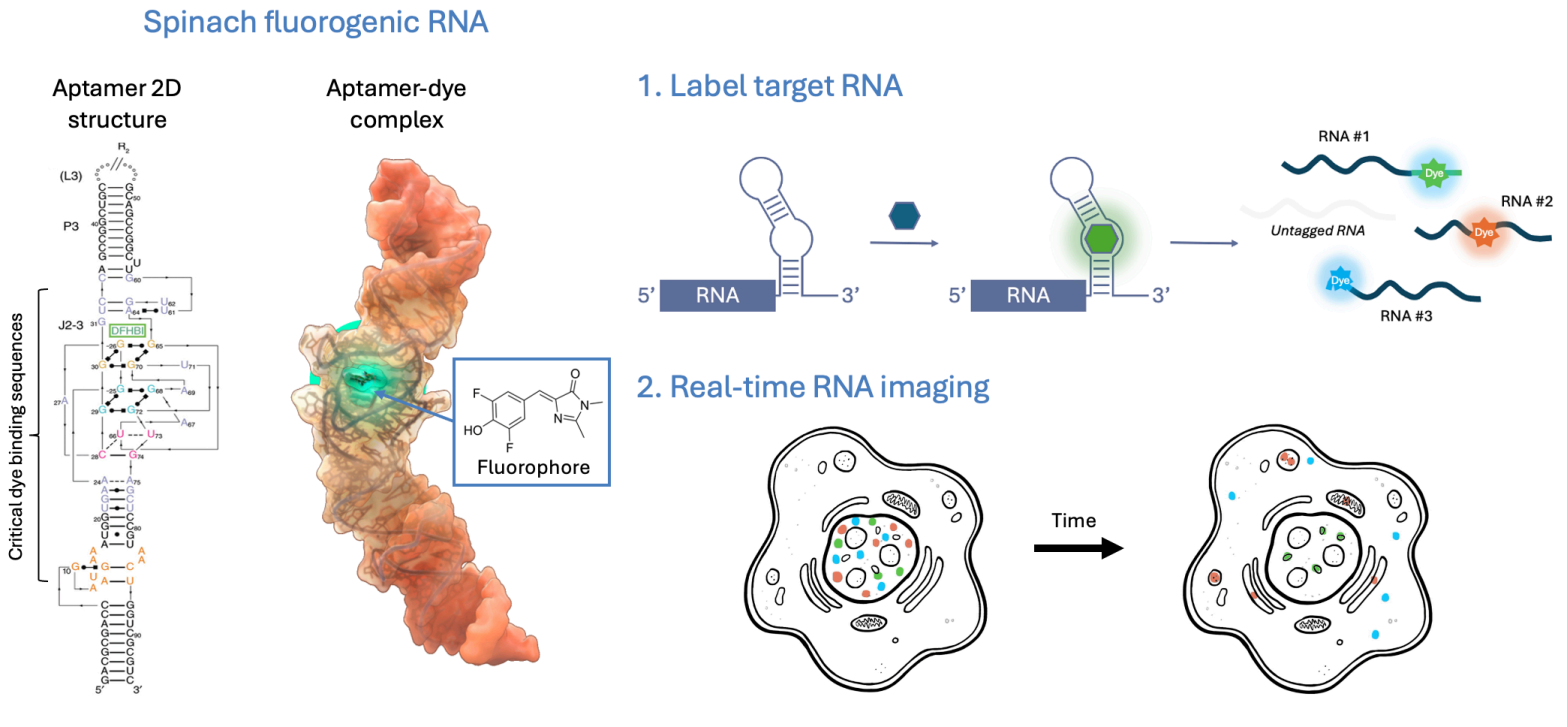


**Graphical representation of the Spinach^TM^ technology principle**

## Background

For decades, cellular complexity was attributed primarily to protein diversity. It is now clear that RNA populations are extraordinarily diverse, encompassing not only mRNA, rRNA, and tRNA but also numerous noncoding classes, including long noncoding RNAs (lncRNAs), microRNAs, PIWI-interacting RNAs, circular RNAs, and more [1]. Current human genome annotations list ~19–20k protein-coding genes versus >35k lncRNA genes, underscoring that annotated noncoding genes now outnumber protein-coding genes [2]. Further, thousands of small noncoding RNAs are also cataloged. Emerging work continues to define additional small-RNA subclasses, their biogenesis, and regulatory roles across physiology and disease.

RNA fate is shaped by compartmentalized processing events—capping, splicing, editing, modification, decay, and localization—many of which occur in, or are regulated by, nuclear and cytoplasmic ribonucleoprotein condensates (e.g., nucleolus, Cajal bodies, nuclear speckles, and paraspeckles) [3]. These bodies depend on RNA for integrity and function and are increasingly linked to human disease. In the nervous system, RNA localization and local translation in axons, dendrites, and synapses support development and plasticity, making spatial RNA regulation particularly consequential [4,5]. Techniques that resolve RNA dynamics in living cells are therefore essential for connecting localization and processing to function.

Genetically encodable fluorescent proteins transformed cell biology by enabling routine, live-cell visualization of protein localization and trafficking. Analogous tools for RNA have matured substantially, but each strategy carries distinct trade-offs that must be considered when selecting methods for live-cell imaging.

Quenched hybridization probes (molecular beacons) directly target endogenous transcripts and provide fluorogenic turn-on and spectral multiplexing, but face delivery and nuclear sequestration issues and require target-specific designs [6]. Protein-recruitment tags (MS2/PP7 stem-loops with coat proteins) remain a widely used approach for single-mRNA tracking and are compatible with genome editing. However, these systems add large RNA payloads, introduce background signals from unbound coat proteins, and can perturb RNA biology through altered localization or impaired stability [7,8]. CRISPR–dCas13 imaging enables programmable detection of endogenous RNAs, yet its application is limited by the large protein cargo and potential off-target effects, and can introduce fluorescence from unbound complexes [9,10].

Fluorogenic RNA aptamers (also called fluorescence light-up aptamers) are short, structured RNA sequences that bind non-fluorescent small-molecule dyes and activate their fluorescence upon binding. Aptamer-dye pairs such as Spinach^TM^ family, Mango, and Peppers provide genetically encodable, compact tags with high signal-to-background contrast and expanding orthogonal palettes that enable powerful multiplex imaging. Compared with protein-based or probe-based approaches, fluorogenic aptamers offer smaller genetic footprints, direct integration into native transcripts, and highly versatile applications from single-molecule tracking to high-throughput assays. Although aptamer folding efficiency remains a technical concern, cellular perturbations have not been widely reported, and careful design can further minimize risks of altering RNA biology. Importantly, the high specificity of fluorophore binding arises from unique three-dimensional aptamer folding that creates well-defined binding pockets, thereby preventing interaction with unrelated structured RNAs and minimizing off-target effects [11,12]. With ongoing engineering to improve folding robustness, labeling efficiency, and dye performance, fluorogenic RNA aptamers are rapidly emerging as one of the most versatile and biologically compatible tools for live-cell RNA imaging.

This protocol provides a focused guide to the use and applications of the Spinach^TM^ family of fluorogenic RNA aptamers. Spinach was among the first genetically encodable RNA aptamers to enable robust fluorogenic imaging in living cells [13]. Here, we use “Spinach^TM^ family” as an umbrella term for Spinach and related improved aptamers (e.g., Broccoli, Corn, Squash, Beetroot), together with their cognate small-molecule fluorogens (e.g., DFHBI, DFHBI-1T, DFHO, BI). These fluorophores are cell-permeable and, when bound by their matching aptamers, become highly fluorescent with minimal background in cells. In the absence of the aptamer, they exhibit little to no fluorescence, enabling high contrast. As genetically encodable tags, Spinach^TM^ family aptamers have been widely used to interrogate RNA localization, dynamics, and regulation—both from exogenous constructs and at endogenous loci—across diverse biological and disease contexts [14–22].

Members of the Spinach^TM^ aptamer family are named for vegetables whose colors mirror their emission (e.g., Spinach/Broccoli: green; Corn: yellow; Squash: orange; Beetroot/Red Broccoli: red). This convention echoes the color-themed, fruit-inspired nomenclature popularized for fluorescent protein variants.

In this protocol, we describe step-by-step how to generate Spinach^TM^-tagged RNA constructs and express them in mammalian cell lines, acquire live-cell imaging, and analyze the resulting data. We also present selected application examples that highlight how these tools can be deployed across varied cellular applications.

## Materials and reagents

**Biological materials**

1. HeLa human cervical epithelial cells (ATCC, catalog number: CRM-CRL-2)

2. HEK293T human kidney epithelial cells (ATCC, catalog number: CRL-3216)

3. COS-7 monkey fibroblasts (ATCC, catalog number: CRL-1651)

**Reagents**

1. DMEM, high glucose, GlutaMax^TM^, pyruvate, phenol red (ThermoFisher, catalog number: 10569010)

2. Fetal bovine serum (FBS) (ThermoFisher, catalog number: 26140079)

3. Penicillin-streptomycin solution (pen-strep) (ThermoFisher, catalog number: 15140122)

4. TrypLE^TM^, no phenol red (ThermoFisher, catalog number: 12563011)

5. GlutaMax^TM^ supplement (ThermoFisher, catalog number: 35050061)

6. Cell culture-grade water (Millipore Sigma, catalog number: W3500-6X500ML)

7. Phosphate-buffered saline, 1× (PBS) (Corning, catalog number: 21-040-CV)

8. Trypan Blue, 0.4% solution (Thomas Scientific, catalog number: C838W29)

9. Poly-L-lysine solution, 0.1 mg/mL (PLL) (Millipore Sigma, catalog number: A-005-C)

10. Human plasma fibronectin, 5 mg (Corning, catalog number: 356008)

11. FuGENE^®^ HD (Promega, catalog number: E2311)

12. Opti-MEM (ThermoFisher, catalog number: 31985062)

13. FluoroBrite^TM^ DMEM (ThermoFisher, catalog number: A1896702)

14. Hoechst 33342, 10 mg/mL (ThermoFisher, catalog number: H3570)

15. SYTOX^TM^ red dead cell stain, 5 μM (ThermoFisher, catalog number: S34859)

16. Plasmids:

a. pcDNA3.1-mCherry-48xBroccoli-polyA_150 _(generated in-house)

b. pAV-U6-Corn (Addgene, catalog number: 106233)

c. pAV-U6-F30-2xdBroccoli (Addgene, catalog number: 66842)

d. pAV-U6-Squash (Addgene, catalog number: 177913)

e. pcDNA3.1-MAPT-4xSquash (generated in-house)

f. pcDNA3.1-CUG_12_-2xSquash (generated in-house)

g. pcDNA3.1-CUG_240_-2xSquash (generated in-house)

h. pcDNA3.1-CGG_60_-Spinach2 (generated in-house)

i. pEGFP (Addgene, catalog number: 165830)

17. BI fluorophore (Lucerna, catalog number: 600-1mg)

18. DFHO fluorophore (Lucerna, catalog number: 500-1mg)

19. Dimethyl sulfoxide, cell culture grade (DMSO) (VWR, catalog number: IC0219605580)

20. Nikon Immersion Oil Type F, N = 1.516 (Morrell Instrument Co., catalog number: MXA22168)

21. HiScribe^®^ T7ARCA mRNA kit (NEB, catalog number: E2060S)

22. RNA clean & concentrator (Zymo, catalog number: R1018)

**Solutions**

1. Cell culture media (see Recipes)

2. Fibronectin stock solution (see Recipes)

3. BI fluorophore stock solution (see Recipes)

4. DFHO fluorophore stock solution (see Recipes)

5. 1× live-cell imaging solution (see Recipes)

**Recipes**

**1. Cell culture media**

**Table:** 

Reagent	Final concentration	Quantity or volume
DMEM	89%	445 mL
FBS	10%	50 mL
Pen-strep	1%	5 mL
Total		500 mL

**2. Fibronectin stock solution**

**Table:** 

Reagent	Final concentration	Quantity or volume
Human fibronectin	1 mg/mL	5 mg
Sterile ddH_2_O		5 mL
Total		5 mL

*Note: Store fibronectin stock solutions in 50 μL aliquots at -80 °C. Avoid multiple freeze-thaw cycles.*

**3. BI fluorophore stock solution**

**Table:** 

Reagent	Final concentration	Quantity or volume
BI fluorophore (M.W. 368.3)	40 mM	1 mg
DMSO		68 μL

*Note: Store BI stock solutions at -20 °C in the dark.*

**4. DFHO fluorophore stock solution**

**Table:** 

Reagent	Final concentration	Quantity or volume
DFHO fluorophore (M.W. 281.2)	40 mM	1 mg
DMSO		90 μL

*Note: Store DFHO stock solutions at -20 °C in the dark.*

**5. 1× live-cell imaging media**

**Table:** 

Reagent	Final concentration	Quantity or volume
FluoroBrite DMEM	1×	499.4 μL
Hoechst 33342, 10 mg/mL	5 μg/mL	0.25 μL
SYTOX red dead cell stain, 5 μM	2.5 nM	0.25 μL
Fluorophore stock solution*, 40 mM	10 μM	0.125 μL
Total		500 μL/well

*Note: 0.5 mL is sufficient for one well. Determine the total volume needed by multiplying the total number of wells needed by 0.5 mL and adding a 10% excess.*

*Fluorophore(s) selection should correspond to the Spinach^TM^ tag(s) used (see Table 1). If multiplexing, two spectrally separated fluorophores should be used (e.g., Broccoli and Squash).

**Table 1.** Properties of Spinach^TM^ tags

Spinach^TM^ tag	Length (nt)	Fluorophore	Ex (nm)	Em (nm)	Kd (nM)	Note
Broccoli	49	BI	470	505	51	Multiplex w/ squash, photostable
Spinach2	95	DFHBI-1T	482	505	560	Ideal for bacterial imaging
Broccoli	49	DFHBI-1T	472	507	305	Bacterial, in vitro, cell imaging
Corn	28	DFHO	505	545	70	Forms dimer, can induce condensate
Squash	71	DFHO	495	562	58	Multiplex w/ broccoli, photostable
Red Broccoli	54	OBI	541	590	23	
Beetroot	37	DFAME	514	619	460	Forms dimer, can induce condensate
Squash	62	DFQL-1T	516	654	56	Tissue imaging capability

**Laboratory supplies**

1. Glass-bottom, 24-well plate (VWR, catalog number: 82050-898) or 4-well dish (VWR, catalog number: 890135-976)

2. Microcentrifuge tubes, 1.5 mL (USA Scientific, catalog number: 1615-5500)

3. Sterile centrifuge tube, 15 mL (CELLTREAT, catalog number: 667015B)

4. Sterile centrifuge tube, 50 mL (CELLTREAT, catalog number: 229421)

5. Parafilm (Bemis, catalog number: PM999)

6. T-75 flasks w/ filter cap (USA Scientific, catalog number: CC7682-4875)

7. Serologic pipette, 2 mL individually wrapped (Fisher, catalog number: 13-678-11C)

8. Serologic pipette, 5 mL individually wrapped (Fisher, catalog number: 13-678-11D)

9. Serologic pipette, 10 mL individually wrapped (Fisher, catalog number: 13-678-11E)

10. Serologic pipette, 25 mL individually wrapped (Fisher, catalog number: 13-678-11)

11. Serologic pipette, 50 mL individually wrapped (Fisher, catalog number: 13-678-11F)

12. Pipette tips w/ filters, P2 (Denville, catalog number: 1159M41)

13. Pipette tips w/ filters, P20 (Denville, catalog number: 1159M43)

14. Pipette tips w/ filters, P200 (Denville, catalog number: 1159M40)

15. Pipette tips w/ filters, P1000 (Denville, catalog number: 1159M42)

## Equipment

1. Certified type A2 biosafety cabinet (VWR, catalog number: 89413-128)

2. Pipette, single-channel P2 (Eppendorf, model: Research Plus)

3. Pipette, single-channel P20 (Eppendorf, model: Research Plus)

4. Pipette, single-channel P200 (Eppendorf, model: Research Plus)

5. Pipette, single-channel P1000 (Eppendorf, model: Research Plus)

6. Pipette fillers (ThermoFisher, model: S1 pipet fillers)

7. 4 °C refrigerator (Summit, model: FF7BIADA)

8. -40 °C ultra-low temperature freezer (VWR, catalog number: 76307-990)

9. -80 °C ultra-low temperature freezer (ThermoFisher, model: RLE50086A)

10. Water jacket tissue-culture incubator (VWR, model: 3015)

11. Biological microscope (Bioimager, model: BIM500FL)

12. Cell counter (Corning, catalog number: 6749)

13. Inverted wide-field fluorescence microscope (Nikon, model: Eclipse Ti-E)

14. Microcentrifuge (Eppendorf, model: 5424)

15. Centrifuge (Eppendorf, model: 5702R)

16. Cell counter (Corning, model: 6749)

17. Counting chamber (Corning, catalog number: 480200)

18. Water bath, 37 °C (Fisher Scientific, catalog number: 15-460-10)

19. Metallic water bath beads (Thomas Scientific, catalog number: 1220U61)

20. Stage top environmental chamber with flow control (Tokai Hit, model: INUTIZ)

21. Motorized stage (Nikon, model: TiSER)

22. High-numerical aperture (high-NA) fluorescence objective (Nikon, catalog model: CFI Plan Lambda Apo, 60×/1.4, oil)

23. sCMOS camera (Andor Technology, model: Zyla 5.5)

24. For imaging Hoechst 33342: DAPI filter cube with a sputter-coated excitation filter 395/25, dichroic mirror 425 (beamsplitter), and emission filter 460/50 (Chroma, model: 49028 ET)

25. For imaging Broccoli: FITC/GFP filter cube with a sputter-coated excitation filter 470/40, dichroic mirror 495 (beamsplitter), and emission filter 525/50 (Chroma, model: 49002 ET)

26. For imaging Corn and Squash: YFP filter cube with a sputter-coated excitation filter 500/20, dichroic mirror 515 (long pass), and emission filter 535/30 (Chroma, model: 49003 ET)

27. For imaging Squash: TRITC filter cube with a sputter-coated excitation filter 555/23, dichroic mirror 574 (beamsplitter), and emission filter 609/54 (Semrock, model: LED-TRITC-B-000)

28. For imaging mCherry: DsRed filter cube with a sputter-coated excitation filter 545/30, dichroic mirror 570 (long pass), and emission filter 620/30 (Chroma, model: 49005 ET)

29. For imaging SYTOX^TM^ red: Cy5 filter cube with a sputter-coated excitation filter 620/60, dichroic mirror 660 (long pass), and emission filter 700/75 (Chroma, model: 49006 ET)

## Software and datasets

1. ApE plasmid editor (https://jorgensen.biology.utah.edu/wayned/ape/) [23]

2. NUPACK nucleic acid analysis and design software (https://www.nupack.org/) [24]

3. UNAFold package (https://www.unafold.org/)

4. NIS Elements Advanced Research image acquisition software with High Content Analysis module (Nikon, version 5.4). Free alternative: ImageJ image analysis software with Fiji package (https://fiji.sc/), or similar image analysis software [25]

5. Prism graphing and statistical software (GraphPad, version 10). Free alternative: ggplot2 (https://ggplot2.tidyverse.org/) [26]

## Procedure

**A. Design and generation of Spinach^TM^-tagged RNA expression constructs**

1. Select an aptamer–dye pair. Use Table 1 to choose the RNA aptamer/fluorophore best suited to your goals (e.g., cell type, available filter sets, multiplexing needs).

2. Plan tag placement. Spinach^TM^ tags have been widely used to label diverse RNAs [14–21]. In most cases, tags are inserted in the 5′ UTR and/or 3′ UTR of the target RNA. Published Spinach^TM^ tag sequences are listed in Table 2. Model the sequence insertion in a plasmid editor (e.g., ApE, SnapGene) to ensure it does not disrupt essential elements or functions.

• If inserting in the 5′ UTR: Avoid introducing a start codon; do not interrupt the Shine–Dalgarno sequence in bacteria; do not impede eukaryotic ribosomal scanning.

• If inserting in the ORF: Avoid creating premature termination signals or altering the encoded amino acid sequence.

• If inserting in the 3′ UTR (after the stop codon): Do not disrupt transcription termination or trigger unintended RNA degradation.

• If critical RNA structures are required for function, evaluate the full chimeric sequence in an RNA folding program (e.g., mFold, NUPACK) to check for misfolding.

**Table 2.** Spinach^TM^ tag sequences

Spinach^TM^ tag	Sequence
Beetroot	GTTAGGCAGAGGTGGGTGGTGTGGAGGAGTATCTGTC
Broccoli	GAGACGGTCGGGTCCAGATATTCGTATCTGTCGAGTAGAGTGTGGGCTC
Corn	CGAGGAAGGAGGTCTGAGGAGGTCACTG
Red Broccoli	GAGACGGTCGGGTCCAGTCCCAACGATGTTGGCTGTTGAGTAGTGTGTGGGCTC
Spinach2	GATGTAACTGAATGAAATGGTGAAGGACGGGTCCAGTAGGCTGCTTCGGCAGCCTACTTGTTGAGTAGAGTGTGAGCTCCGTAACTAGTTACATC
Squash	AGGTGAGCCCAATAATACGGTTTGGGTTAGGATAGGAAGTAGAGCCGTAAACTCTCTAAGCG
Scrambled	GATAGGCTAAAACTGAGAGAGAAACTGTAGTTTTGTAGCATAATGCACTGCTGCCAGGGCGG

3. Choose an expression system. Select a vector that can express the Spinach^TM^-tagged RNA under the desired promoter:

• Eukaryotic RNA polymerase II (e.g., CMV, CAG, EF1α) for mRNA expression

• Eukaryotic RNA polymerase III (e.g., 5S, U6, tRNA) for ncRNA expression

• Prokaryotic RNA polymerase promoters (e.g., T7, SP6, lac) for bacterial expression

• See the Addgene promoter overview for additional guidance: https://tinyurl.com/48ntd9ue.

4. Construct generation. Build Spinach^TM^-tagged RNA expression plasmids using standard molecular cloning. Include a negative-control construct in which the Spinach^TM^ tag is replaced with a non-fluorophore-binding sequence of similar length (e.g., a scrambled insert).

5. Sequence verification. Confirm correct insertion by DNA sequencing.

6. DNA preparation for transfection. From a sequence-verified single colony, purify plasmid DNA with a chromatography kit matched to the required yield/purity. Use endotoxin-free formats for mammalian transfection.

**CRITICAL:** Create a bacterial glycerol stock for each sequence-verified plasmid for long-term storage and easy propagation. See Addgene for a bacterial glycerol stock protocol: https://tinyurl.com/7ckajfx9.

**PAUSE POINT:** Purified plasmids can be stored at -20 °C until ready to use.

**B. Preparation of extracellular matrix (ECM) coated glass-bottom plates**

**General consideration:** ECM is a non-cellular network of proteins and carbohydrates that improves the attachment and spreading of adherent cells on glass-bottom plates or dishes. Common ECMs include fibronectin, collagen, poly-L-lysine (PLL), and laminin. Optimal conditions should be determined empirically for each cell line and application.

**CRITICAL:** Perform all sterile cell–related work in a certified biosafety cabinet using proper aseptic technique. Additionally, glass-bottom plates or dishes are required for fluorescence imaging since they provide the necessary optical clarity and flatness for high-numerical-aperture objectives, while plastic-bottom dishes can cause optical distortion, reduced resolution, and compromise image quality.

1. PLL coat. Add 0.5 mL of PLL to each glass-bottom well and incubate for ≥2 h at 37 °C or overnight at 4 °C.

• Wells in 24-well plates and 4-well dishes each hold ~0.5 mL.

• Multiple plates may be coated in parallel. Wrap in sterile parafilm and store at 4 °C.

• PLL can be reused at least three times; store used PLL at 4 °C.

2. Rinse. Aspirate PLL and rinse wells three times with 0.5 mL of ddH_2_O.

**PAUSE POINT:** PLL-coated plates (either dried or wet) can be stored for up to 24 h at room temperature or up to 1 week at 4 °C. Unused wells can be used in future experiments.

3. Sterilize. Remove residual water, leave plates uncovered in the hood, and UV-irradiate for ≥15 min.

4. Prepare fibronectin. Dilute thawed 1 mg/mL stock 1:50 in PBS to make a 20 μg/mL working solution; prepare sufficient volume for 0.3 mL per well.

5. Fibronectin coat. Add 0.3 mL of 20 μg/mL fibronectin to each PLL-coated well and incubate for ≥2 h at 37 °C.

6. Dry. Aspirate fibronectin completely and allow wells to dry. Plates are ready for cell plating.

**CAUTION:** Remove excess fibronectin thoroughly, as residual fibronectin can impair cell health.

**C. Transfection of adherent mammalian cells**

1. Subculture adherent cells at passages ~P5–P20 when cultures reach ~80% confluency.

**CRITICAL:** Passage at regular intervals to maintain reproducible behavior. Adjust seeding density until growth rate and yield are consistent. Plan experiments around the subculture schedule when possible.

2. When the new passage reaches ~80% confluency, dissociate HeLa cells with TrypLE following standard adherent-cell procedures. Determine cell concentration, manually or with an automated counter, using Trypan Blue.

3. Determine the total number of wells needed and prepare a cell suspension sufficient to dispense 0.5 mL per well. Seed enough cells to ensure that cells will reach 60%–80% confluency at the time of transfection.

**Figure 1.**
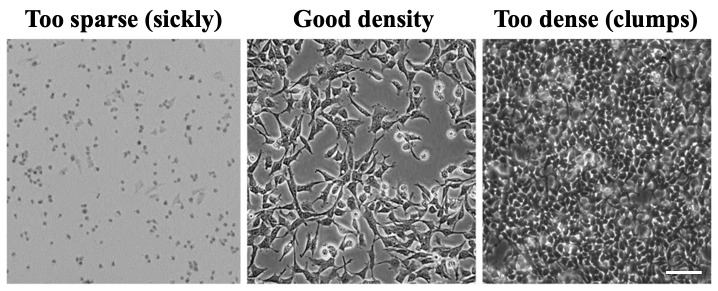
Representative cell density. Cells seeded too sparsely show poor spreading and stressed morphology. Cells at optimal density form an even, well-spread monolayer with minimal overlap. Cells seeded too densely cluster into clumps and multilayer islands, which impairs transfection and imaging. Scale bar: 100 μm.

**CRITICAL:** Optimal density is cell line–specific. Titrate seeding density to achieve healthy growth in the presence of transfection reagents, with full spreading and minimal clumping at 24 h post-transfection (Figure 1).

4. After 24 h, prepare the transfection mix according to the FuGENE^®^ HD protocol: 0.5 μg of Spinach^TM^-tagged RNA expression construct, 1.5 μL of FuGENE^®^ HD, and Opti-MEM to a final volume of 40 μL per well.

• Always include a scrambled aptamer control or negative expression control (FuGENE^®^ HD + Opti-MEM only). In the presence of fluorophores, these controls can be used to measure background fluorescence.

• In separate wells, 0.1 μg of pEGFP may be used as a transfection control, and 0.5 μg of plasmids expressing aptamers alone (pAV-U6-Corn, pAV-U6-F30-2xdBroccoli, pAV-U6-Squash) can be used as positive aptamer controls.

• To save time, reverse transfection can be performed the day of cell plating. But testing should be done prior to the experiment to confirm that reverse transfection did not reduce cell viability of the cell line. A protocol for reverse transfection with FuGENE^®^ HD can be found at https://tinyurl.com/4s2aprw5.

**CRITICAL:** The amount of plasmid DNA and transfection reagent used should be empirically determined to obtain optimal expression and a high signal-to-background ratio.

**D. Live-cell imaging of Spinach^TM^-tagged RNAs**

**General consideration:** Imaging experiments described here were performed on a Nikon Eclipse Ti-E inverted fluorescent microscope in a motorized temperature-controlled chamber. Equivalent inverted fluorescence systems with comparable sensitivity and environmental control are also suitable. For the acquisition steps described below, use the corresponding controls in the software/hardware for your imaging system.

1. Image cells ~24–48 h post-transfection.

*Note: The optimal imaging time depends on the expression kinetics of the tagged RNA (promoter strength, cell type, RNA stability). Run a brief time-course and adjust earlier (6–12 h) or later (up to ~72 h) as needed.*

2. Prepare 1× live-cell imaging medium 1 h before imaging and keep at 37 °C. For extended time-lapse experiments (>6 h), add 10% FBS and 1% GlutaMAX^TM^ to support cell health.

3. Replace culture medium with prewarmed imaging medium 30 min before imaging. Tilt the plate ~45° and gently aspirate without touching cells. Add 0.5 mL of imaging medium dropwise.

4. Pre-equilibrate the microscope environmental chamber to 37 °C and 5% CO_2_ for ≥30 min.

5. Place the plate in the chamber. Add immersion oil to the 60× objective and focus on adherent cells using phase illumination.

*Note: Use immersion oil that is suited for fluorescence microscopy and compatible with the objective used.*

6. In the software, access the acquisition settings menu by clicking *View > Acquisition Controls > Acquisition*.

7. Switch to the DAPI filter to begin fluorescence acquisition. Determine the proper exposure time for each channel by acquiring images from 100 ms to 2 s exposure time. The optimal exposure time should allow the highest possible signal without any pixel saturation.

8. Optimize exposure for all required filter sets (e.g., FITC/GFP, YFP, TRITC, Cy5).

**CRITICAL:** To minimize photobleaching/phototoxicity, limit light between acquisitions, lengthen intervals as needed, and, when possible, acquire from longer to shorter wavelengths (red → yellow → green).

9. Access the ND Acquisition menu by clicking *Applications > Define/Run ND Acquisition*. This feature allows for automated acquisitions with defined time points, defined positions in the well, simultaneous acquisition of any fluorescent channels and brightfield, and the ability to stitch adjacent fields in one image.

10. In the “λ” tab, select filters based on the Spinach^TM^ tag and any co-labels:

• Cy5 filter: To visualize dead cells with SYTOX^TM^ red stain, suggested exposure 300 ms to 1 s.

• TRITC filter: To visualize Squash-tagged RNA, suggested exposure 300 ms to 1 s.

• YFP filter: To visualize Corn-tagged RNA, suggested exposure 300 ms to 1 s.

• FITC/GFP filter: To visualize Broccoli-tagged RNA, suggested exposure 300 ms to 1 s.

• DAPI filter: To visualize Hoechst-stained nuclei, suggested exposure 100–200 ms.

*Note: DAPI stain is not recommended for live-cell imaging due to low permeability and cell toxicity issues.*

11. (Optional) In the “λ” tab, also check the box for *Close Active Shutter during Filter Change* and *Use PFS*. This will help prevent bleedover of light when switching fluorescent channels and help with maintaining accurate overlays of images in different channels during acquisition.

*Note: Perfect focus system (PFS) is a hardware/software Nikon component that continuously maintains focus during live imaging by detecting the coverslip interface with infrared light and automatically correcting Z-drift in real time.*

12. (Optional) In the *Large Image* tab, select the following settings to create a large, stitched image:

• Scan area: 7 × 7

• Overlap: 15%

• Stitching via: Blending

• Check the boxes for *Close Active Shutter during Stage Movement* and *Use PFS*.

*Note: This step is only necessary if taking many neighboring images for quantification purposes.*

13. For each well, select 2–3 fields near the center with adequate cell density (at least 2 cells per 60× field) and clearly defined cell borders. Acquire brightfield and fluorescence images using *ND Acquisition*.

## Data analysis

The NIS Elements Advanced Research image acquisition software includes modules to preprocess images, export TIFF (.tif) images, and quantify fluorescence at defined subcellular locations. With the General Analysis 3 (GA3) module, signals within the correct subcellular locations can be masked and analyzed to obtain mean fluorescence intensity per cell. Alternatively, exported .tif images can be quantified in Fiji or ImageJ software using the *Freehand selection* tool as described in [27]. Group comparisons can be evaluated with a two-tailed t-test (two groups) or one-way ANOVA followed by Tukey’s multiple comparisons test (>2 groups) to determine statistical significance with a P < 0.05 threshold using the Prism software. If multiple images of the same field were acquired over a time course, these values can be used to measure stability and half-life of RNA transcripts and aggregates.

**A. Background subtraction using NIS Elements software**

**Figure 2.**
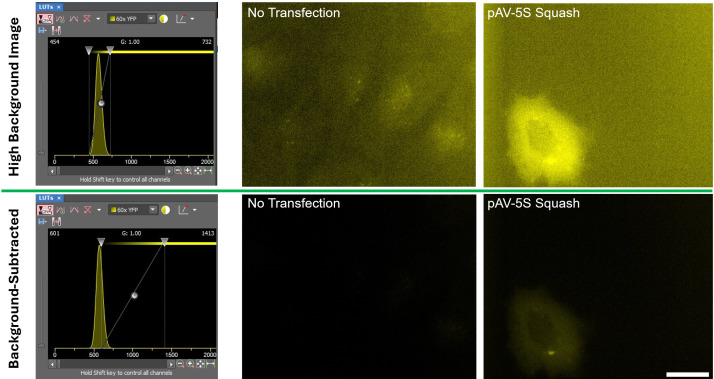
Background subtraction. Cells expressing Squash from the pAV-5S-Squash vector or a negative expression control were imaged with the YFP filter set. The top panels show LUT settings that yield high background (left) and the corresponding images (middle and right). The bottom panels show LUT settings that remove background (left) and the corresponding background-subtracted images (middle and right). By moving the left slider partway into the histogram peak and the right slider further to the right, background-subtracted images can be obtained for any high-background datasets. Scale bar = 20 µm.

**Figure 3.**
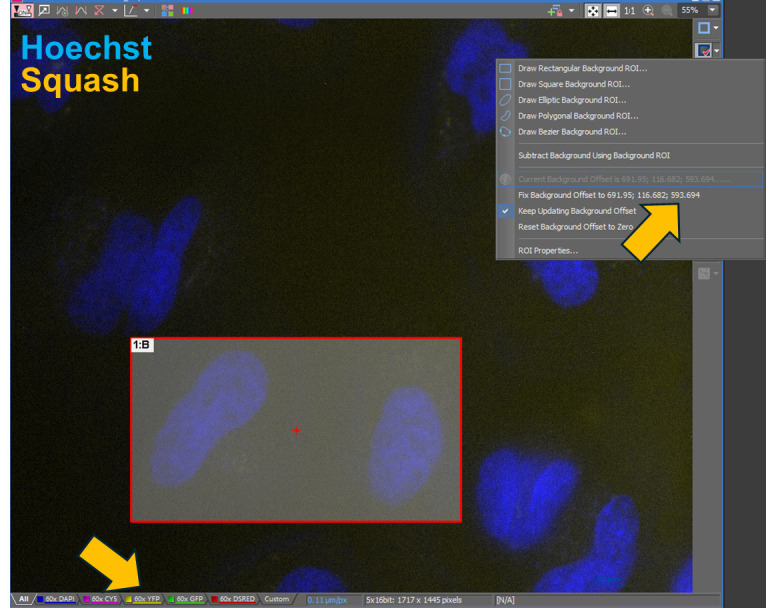
Determining a background constant. This example illustrates how to measure a background constant for GA3 when quantifying Squash fluorescence. The image is from a negative expression control sample with no Squash fluorescence. In the *Turn Background ROI On/Off* menu, draw a *Rectangular Background ROI* over Squash-negative cells. After defining the ROI, retrieve the background constant from the *Fix Background Offset…* option for each channel. Use this value only in the *SubtractConstant* function for the fluorescent channel that requires background subtraction for quantitative analysis.

1. With the negative expression control images for each fluorophore open, access the LUT panel by clicking *View > Image > View LUTs*.

2. For each fluorescent channel, move the left LUT slider to the left half of the histogram peak and raise the right LUT slider until fluorescence within cells is no longer visible (Figure 2).

3. Save these settings, apply them to the remaining images, save the .nd2 files with these LUTs, and export images as .tif files. If the background subtraction is done correctly, the mean intensity of fluorophore-negative cells will be ~0 when measuring this in Fiji or ImageJ software.

4. If the fluorescence intensity is to be measured with GA3, a constant for background subtraction must be determined using the *Turn Background ROI On/Off* function, and then recording the *Background Offset* value (Figure 3).

**CRITICAL:** LUT changes affect display scaling. Always retain raw data for auditing.

**B. Calculate mean fluorescence intensity per cell with GA3**

GA3 is a highly customizable image analysis software that can be tailored to measure fluorescent events based on size, shape, intensity, and subcellular localization. This allows for quick and more unbiased measurement of fluorescence intensity of fluorogenic RNA aptamers over many fields and large stitched images. However, the GA3 recipes are complicated and lengthy. A summary of the functions needed to extract a mean fluorescence intensity from images will be described instead. Similar functions can be found on Fuji/ImageJ as well. A tutorial for analyzing images on GA3 and exporting the mean fluorescence intensity data can be found at https://www.youtube.com/watch?v=k6TopfhQzPk.

**Figure 4.**
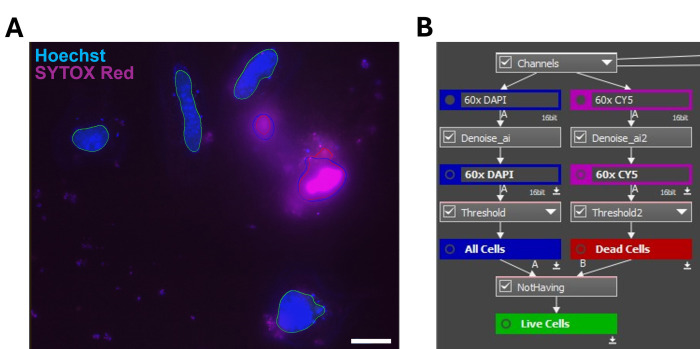
Excluding dead cells from analysis. An example of using masking in GA3 to distinguish live and dead cells. (A) During analysis, nuclei masks from cells stained with Hoechst (blue) are colocalized with nuclei masks of dead cells stained with SYTOX^TM^ Red (red). Overlapping masks identify dead cells to be excluded, while nuclei in the green masks represent live cells that will be used for further analysis. Scale bar = 20 µm. (B) Schematic of the GA3 workflow to highlight live cells. *Denoise_ai* helps maximize the signal-to-background, *Threshold* is the function for masking positive fluorescent events, and *Not Having* excludes colocalized masks.

**Figure 5.**
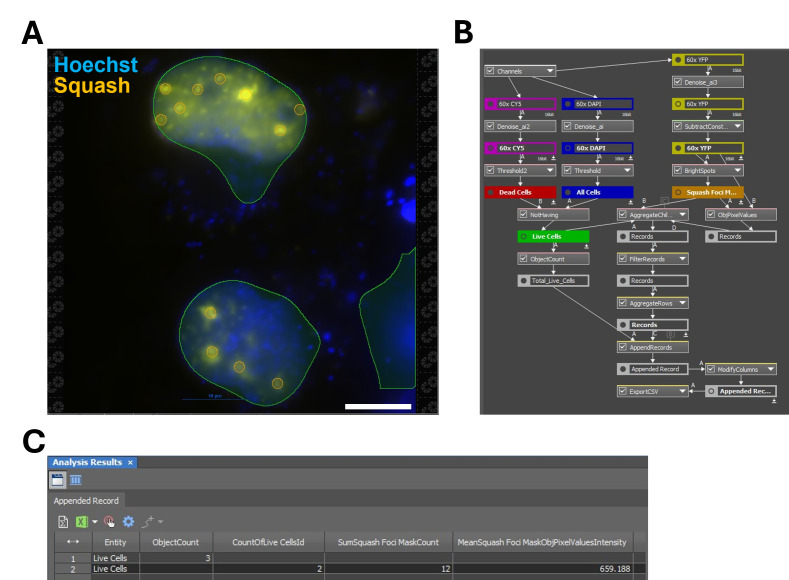
Measuring a mean fluorescence intensity for fluorescent RNA foci. Images of COS-7 cells expressing a Squash-tagged 240× CUG repeat RNA were analyzed in GA3 to quantify nuclear foci formation. (A) Example images and GA3 masks. Masks of live-cell nuclei (green) and RNA foci (orange) are overlaid on images acquired with DAPI (Hoechst) and YFP (Squash) channels. Scale bar = 10 µm. (B) Schematic of the GA3 workflow to quantify mean fluorescence intensity of nuclear RNA foci in live cells. Background-subtraction constants are entered in *SubtractConstant*. Foci are detected with the *BrightSpots* function. *ObjPixelValues* calculates per-pixel intensity, and *AggregateChildren* retains pixels that fall within nuclear foci of live cells. *FilterRecords* sets the minimum foci-per-cell threshold for inclusion. Background-only cells typically have fewer than four foci. (C) Representative output table. *ObjectCount* is the total number of live cells, *CountOfLive CellsId* is the number of cells meeting the minimum foci threshold, *SumSquash Foci MaskCount* is the total number of fluorescent nuclear foci, and *MeanSquash Foci MaskObjPixelValuesIntensity* is the mean fluorescence intensity of the RNA foci for the sample.

**Figure 6.**
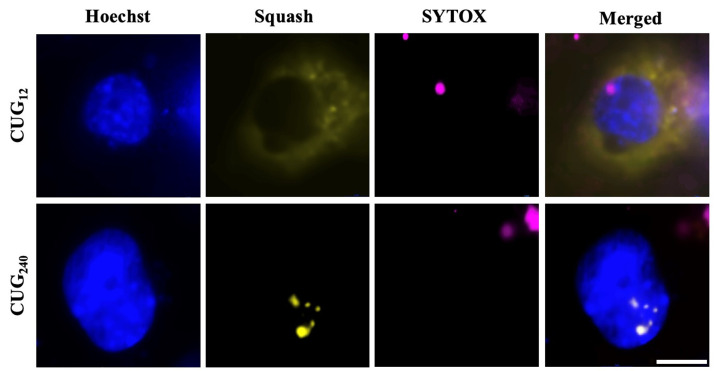
Expanded Squash-tagged CUG repeats form large bright RNA foci in the nucleus. To compare normal and pathogenic repeat lengths, plasmids encoding Squash-tagged *DMPK* 3′-UTR constructs bearing 12 (CUG12) or 240 (CUG240) repeats were transfected into cells (pcDNA3.1-CUG_12_-2xSquash and pcDNA3.1-CUG_240_-2xSquash). In COS-7 fibroblasts loaded with DFHO and imaged using a YFP filter set, CUG_12_ produced diffuse cytoplasmic Squash fluorescence (yellow), whereas CUG_240_ yielded bright nuclear puncta. Nuclei were counterstained with Hoechst 33342 (blue) and nonviable cells with SYTOX^TM^ Red (red). These patterns recapitulate the localization of endogenous *DMPK* transcripts observed by fluorescence in situ hybridization. Scale bar = 10 µm.

1. Open the background-subtracted images of samples with fluorescent cells. Then, open the GA3 menu by clicking *Image > New GA3 Recipe*.

2. Link a *Denoise* function to each fluorescent channel. For the fluorescent channel that will be analyzed quantitatively, link a *SubtractConstant* function to *Denoise*. This function is how GA3 performs background subtraction.

3. Dead cells that are SYTOX^TM^-red-stain-positive (strong red fluorescent signals in the nucleus) can be excluded from analysis with a *Not Having* function (Figure 4).

• Link a *Threshold* function to each of the *Denoise* functions for DAPI and Cy5 channels.

• Within *Threshold*, use the *Intensity, Smooth, Separation, Size*, and *Circularity* functions to create a mask over fluorescent positive pixels.

• Take the thresholded DAPI mask and use *Not Having* for thresholded Cy5 mask. This will create a mask for live cells and exclude dead cells.

4. For measuring fluorescent RNA foci in the nucleus (Figures 5 and 6):

a. Input the value from the *Background Offset* (obtained from Data analysis, step A4) into the *SubtractConstant* function.

b. Small fluorescent foci should be masked with the *Contrast* and *Typical Diameter* functions with *BrightSpots*.

c. Combine the live cell and *BrightSpots* masks using the *Aggregate Children* function, then filter out nuclei with fewer than four foci with the *Filter Records* function. This record will contain the integrated fluorescence intensity for each foci in each cell with more than four foci. Setting a four-foci minimum helps exclude auto-fluorescent cell debris being counted as positive foci.

d. Using the *AggregatedRows* function on this data will yield the mean fluorescence intensity per cell.

**Figure 7.**
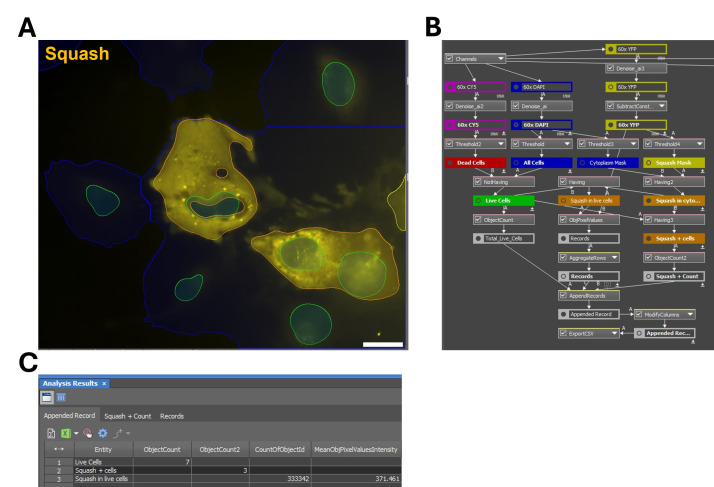
Measuring mean fluorescence intensity for cytoplasmic fluorescent RNA. Images of HeLa cells expressing Squash-tagged MAPT RNA were analyzed in GA3. (A) Example images and GA3 masks. Masks of live cell nuclei (green) and Squash fluorescence (yellow) are overlaid on the YFP (Squash) channel. A cytoplasmic mask derived from the DAPI-thresholding method (blue) and a mask of Squash signal in live cells (orange) are indicated. Scale bar = 20 µm. (B) Schematic of the GA3 workflow to quantify the mean fluorescence intensity of Squash RNA in live cells. One notable difference from Figure 5B is the use of *Having* functions to measure Squash signal specifically within the cytoplasm of live cells. (C) Representative output table. *ObjectCount* is the total number of live cells, *ObjectCount2* is the number of live cells expressing Squash, *CountOfObjectID* counts the total number of Squash-positive pixels, and *MeanObjPixelValuesIntensity* is the mean cytoplasmic Squash fluorescence for the sample.

**Figure 8.**
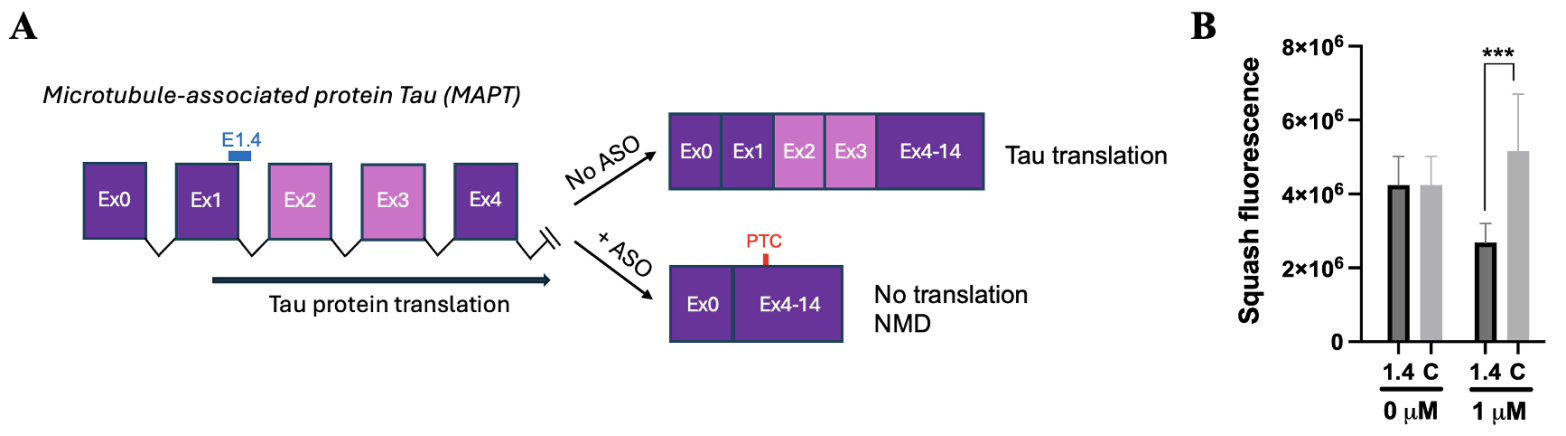
Squash reporter quantified ASO-triggered MAPT transcript NMD levels in cells. (A) *MAPT* contains 16 exons, with exons 2, 3, 6, 8, and 10 (light purple) undergoing alternative splicing. The splicing of exon 2 or exon 3 introduces a premature termination codon (PTC) that triggers NMD. (B) To monitor degradation, a *MAPT* exon 0–4 minigene reporter was constructed with a Squash tag inserted 3′ of exon 4. The reporter (pcDNA3.1-MAPT-4xSquash) was expressed in HeLa cells, and Squash signals were acquired with a YFP filter. E1.4 is an antisense oligonucleotide (ASO) previously shown to induce exon 1 skipping and engage NMD. E1.4 treatment significantly reduced Squash fluorescence, indicating ASO-induced degradation of *MAPT* transcripts. The ASO-induced decreases in *MAPT* transcripts were confirmed by qPCR. Statistical significance was calculated from the Student’s two-tailed t-test. Error bar = SEM, 1.4 is E1.4, and C is no-targeting ASO control. Squash fluorescence is the mean fluorescence intensity per cell for n = 124 cells (0 μM E1.4 and C), n = 16 cells (1 μM E1.4), and n = 102 cells (1 μM C) identified as expressing Squash over four technical replicates.

5. For measuring fluorescent RNA in the cytoplasm (Figures 7 and 8):

a. Input the value from the *Background Offset* (obtained from Data analysis, step A4) into the *SubtractConstant* function.

b. Expression of fluorescent RNA in the cytoplasm tends to yield larger fluorescent objects (>10 μm). These should be masked with the *Intensity, Smooth, Separation, Size*, and *Circularity* functions with *Threshold*.

c. *Threshold* should be used to create a mask over the entire cell in the DAPI channel. Hoechst strongly stains nuclei, but by decreasing the LUT enough to reveal dim signals, it also reveals cell borders. Create a mask with DAPI thresholding comparable to the brightfield image. Verify that a mask with DAPI thresholding matches the brightfield image.

d. Combine the masks using the *Having* function, then measure the total fluorescence of each cell with the *ObjPixelValuesfunction*.

e. Using the *AggregatedRows* function on this data will yield the mean fluorescence intensity per cell.

**C. Using mean fluorescence intensity per cell to measure RNA stability and half-life**

1. Criteria for including mean fluorescence intensity per cell data from samples:

• Within a 7 × 7 field stitched image, GA3 was able to mask and measure at least 20 fluorescent positive cells that were not dead.

• Having data from at least two 7 × 7 field stitched images per well for technical replicates.

• Having two biological replicates.

2. Please refer to [28] for details on making fluorescence-based trajectory plots and calculating RNA half-life. An example of the fluorescence-based RNA half-life plots can be found in Figure 9B.

**D. Application examples**

**1. Imaging of expanded CUG-repeat RNA foci**

Myotonic dystrophy type 1 (DM1) is an autosomal dominant multisystem disorder caused by CTG expansion in *DMPK*. Expanded alleles are transcribed into toxic CUG-repeat RNA. Normal *DMPK* transcripts contain ~10–37 repeats, whereas DM1 alleles produce ~50–5,000 repeats that correlate with earlier onset and greater severity and assemble into nuclear RNA foci. These foci sequester MBNL proteins and disrupt alternative splicing; imaging them enables direct visualization and quantification of the pathogenic inclusions [29].

The RNA-mediated pathogenesis model has been described in other trinucleotide-repeat disorders, such as fragile X-associated tremor/ataxia syndrome (FXTAS), which is caused by expanded CGG repeats. In Strack et al. [27], a Spinach2-tagged CGG_60_ reporter (pcDNA3.1-CGG_60_-Spinach2) was used to image CGG foci dynamics in the presence of two CGG-binding compounds. Tautomycin, but not compound 1a, induced disaggregation of pre-formed CGG foci.

**2. Single-molecule imaging of in vitro–transcribed mRNA in cells**

Single-molecule imaging of in vitro transcribed mRNA in cells enables direct study of mRNA-therapeutic delivery and translation, resolving endosomal escape, cytosolic release, trafficking, and decay in real time.

**3. Imaging of RNA transcription activity in cells**

Imaging RNA transcription in living cells provides a direct, time-resolved view of gene activation, quantifying initiation, pausing, and termination, and enables single-cell assessment of the potency and heterogeneity of transcription activators and inhibitors.

Figure 4A in Song et al. [28] described how the Corn aptamer was inserted into an expression construct under the control of U6 small nuclear RNA (snRNA) promoter. In the presence of the DFHO fluorophore, Corn fluorescence (yellow) reports U6 promoter activity. Corn signals in transfected HEK293T cells transfected with pAV-U6-Corn were recorded over time with a YFP filter set. Actinomycin D (pan-transcriptional inhibitor) produced rapid, global repression of Corn signals, consistent with acute inhibition of U6 promoter output. Figure 5B in Song et al. [28] showed that temsirolimus (mTOR inhibitor) elicited heterogeneous responses, with some cells showing rapid repression and others displaying delayed inhibition.

**4. Imaging of microtubule-associated protein tau (*MAPT*) mRNA degradation**

*MAPT* encodes tau, a microtubule-stabilizing protein essential for neuronal structure and axonal transport. Its dysregulation contributes to neurodegenerative disease. Imaging *MAPT* mRNA degradation provides time-resolved, single-cell readouts of transcript turnover and target engagement by ASOs, siRNA, or nonsense-mediated decay (NMD)-based strategies.

## Validation of protocol

This protocol (or parts of it) has been used and validated in the following research article(s):

• Paige et al. [13]. RNA mimic of green fluorescence protein. *Science* 333, 642 (Figure 4B).

• Li et al. [30]. Fluorophore-promoted RNA folding and photostability enable imaging of single Broccoli-tagged mRNAs in live mammalian cells. *Angew Chem Int Ed Engl* 59(11) (Figures S15 and 16).

• Song et al. [28]. Imaging RNA polymerase III transcription using a photostable RNA-fluorophore complex. *Nat Chem Biol* 13(11) (Figures 4 and 5).

• Strack et al. [17]. A superfolding Spinach2 reveals the dynamic nature of trinucleotide repeat-containing RNA. *Nat Methods* 10(12) (Figures 5 and 6).

## General notes and troubleshooting

**General notes**

1. Always validate the localization and functions of Spinach^TM^-tagged RNAs using established cellular markers or tool compounds.

2. Spinach^TM^-tagged RNA can be delivered into mammalian cells by either transfection or electroporation. Transfection is typically preferred for plasmid constructs because it is simple, equipment-free, and scalable. Electroporation is recommended when the cargo is in vitro–transcribed mRNA or when rapid, transcription-independent expression or difficult-to-transfect cell types are the goal.

3. The Spinach^TM^ family fluorogens are generally cell-permeant in 2D mammalian cultures and produce a fluorogenic signal within minutes to tens of minutes after addition. Permeability and steady-state levels depend on cell type, serum binding, and transporter activity. Consequently, brief titration of dye concentration and incubation time is recommended. For 3D cultures or organoids, slower diffusion and potential efflux can reduce intracellular dye levels. Therefore, longer loading periods, increased dye concentration in the imaging solution, gentle agitation/perfusion, or thinner specimens can improve signal. It is important to monitor for cytotoxicity at higher dye concentrations, and balance wash steps against the need to maintain intracellular dye equilibrium.

**Troubleshooting**

**Problem 1:** High background fluorescence.

**Possible causes:** Suboptimal transfection conditions, dye concentration, or acquisition settings.

**Solution 1:** Optimize the DNA amount and the DNA:FuGENE^®^ HD ratio transfected to increase expression without significantly causing more cytotoxicity. The cell density may need to be adjusted to maximize transfection efficiency and reduce cytotoxicity. Use high-quality, endotoxin-reduced DNA.

**Solution 2:** The dyes themselves may contribute to the background. Consider using 2–5 μM of dyes instead.

**Solution 3:** Optimize the acquisition settings in NIS-Elements. Determine exposure time empirically and avoid pixel saturation. Adjust LUT values to keep the background low while maximizing the signal. Verify that the filter set matches the aptamer–dye pair and rule out bleed-through with single-label controls.

**Problem 2:** Observed high cytotoxicity after transfection.

**Possible cause:** Suboptimal transfection conditions or improper placements of the Spinach^TM^ tags.

**Solution 1:** High concentrations of FuGENE^®^ HD are toxic to cells. Reduce FuGENE^®^ HD to 1–2 μL per 24-well. Cytotoxicity from FuGENE^®^ HD can also be mitigated by transfecting a higher density of cells. Also, consider changing the media 8–24 h post-transfection.

**Solution 2:** Improper placement of the aptamer tag relative to the gene of interest can produce aberrant, cytotoxic RNAs or proteins, especially when overexpressed in cells. If toxicity occurs, test alternative tag locations following step A2 to identify positions that reduce cytotoxicity, or lower the amount of expression vector used.

**Problem 3:** Spinach^TM^-tagged RNA does not show the expected localization or displays unexpected intracellular distribution.

**Possible cause:** The Spinach tag was inadvertently inserted into a critical region.

**Solution:** Double-check literature and perform database search (e.g., RBMmap, Rfam, UTRdb, RegRNA 2.0) to identify potential RNA motifs. Move tags away from known regulatory elements and/or add a neutral linker.

**Problem 4:** Blurry or partially unfocused images during acquisition.

**Possible causes:** PFS focusing issues, insufficient oil on the objective lens, or obstructed automated stage issues.

**Solution 1:** NIS-Elements PFS system autofocuses when moving from field to field during acquisition of a large-stitched image. If the plate is unevenly placed in the chamber or if there is too much debris in the well, the PFS will have trouble autofocusing, resulting in blurry images. Reseat and level the plate and reduce debris with an additional 1× PBS rinse before imaging.

**Solution 2:** Insufficient immersion oil on the objective lens will also cause images to be out of focus. Replenish immersion oil between wells as needed.

**Solution 3:** The automated stage must rapidly move with accuracy to provide clear images between fields. Verify stage movement is smooth and unobstructed. Re-home axes if stitching drifts.

**Problem 5:** Inconsistent fluorescent and cell morphologies during live-cell imaging.

**Possible cause:** Cells are slowly dying during live imaging.

**Solution 1:** Check that the plate chamber is maintaining the 37 °C and 5% CO_2_ conditions over the duration of the imaging acquisition.

**Solution 2:** For extended time-lapse experiments, supplement the live-cell imaging media with 10% FBS and 1% GlutaMAX^TM^ to support cell health.

**Problem 6:** GA3 mean-intensity results do not match images taken.

**Possible cause:** Improper masking was set in GA3.

**Solution:** Validate that the thresholds set on GA3 properly mask the correct fluorescent events over multiple fields and in multiple wells. If there are abundant false negatives, the restrictions on intensity, size, and circularity might be too stringent and should be broadened. Masks that include too much background may be adjusted to have more stringent settings.
